# A223 UNMASKED OR INDUCED COLITIS: COULD IT BE SECUKINUMAB?

**DOI:** 10.1093/jcag/gwac036.223

**Published:** 2023-03-07

**Authors:** S Nassiri, G Ou, W Xiong

**Affiliations:** 1 Gastroenterology; 2 Pathology & Laboratory Medicine, University of British Columbia, Vancouver, Canada

## Abstract

**Background:**

Spondyloarthropathies (SpA), such as Ankylosing Spondylitis (AS) are inflammatory disorders which have coheritability with inflammatory bowel disease (IBD). Secukinumab is a monoclonal antibody inhibitor of IL-17A used to treat SpA, but has also been reported to cause de novo and exacerbations of known IBD. Rare reports of Secukinumab induced microscopic colitis have also been documented.

**Purpose:**

To present and discuss the diagnostic challenges in a case of undifferentiated colitis in a patient with AS treated with Secukinumab.

**Method:**

Case report and literature review.

**Result(s):**

**Case Report:** A 62-year-old male with a history of AS was admitted to hospital with acute on chronic diarrhea. The patient’s AS was unresponsive to Infliximab, Methotrexate, and Adalimumab, although the latter improved his diarrhea at the time.

A Colonoscopy completed 14 years earlier for chronic diarrhea was endoscopically normal with biopsies of the left colon notable for mild patchy inflammation and fibrosis. A subsequent Colonoscopy with random biopsies 8 years later showed normal mucosa while he was treated with Adalimumab.

He was started on Secukinumab 4 months prior to hospitalization noting progressive watery diarrhea and weight loss of 15kg. Investigations were notable for microcytic anemia (Hemoglobin 120 g/L, MCV 78 fL), elevated CRP (33 mg/L), and a creatinine of 508 µmol/L. Negative tTg antibodies and normal IgA levels ruled out celiac disease. Stool studies were unremarkable. Colonoscopy showed several linear ulcerations in the descending colon and rectum with a normal terminal ileum, transverse, and ascending colon. Biopsies from the endoscopically normal mucosa showed thickened subepithelial collagen band suggestive of microscopic colitis while the ulcerated regions showed cryptic rupture with associated granuloma representing possible Crohn’s colitis versus drug-induced injury. Stains were negative for CMV.

Secukinumab was stopped and he was started on Budesonide with significant improvement in symptoms.

**Literature Review:**

Almost half of all patients with SpA have microscopic intestinal inflammation and of these, 7% eventually develop IBD. Shorter symptom duration, higher SpA activity and male sex are known risk factors for underlying intestinal involvement. However, the exact role of microscopic intestinal inflammation in SpA has yet to be determined.

Secukinumab is an inhibitor of IL-17A with significant efficacy in the treatment of SpA but has been associated with exacerbations of Crohn’s disease and de novo cases of IBD. Thus, the role of IL-17 in IBD remains uncertain and may suggest a protective effect rather than an inflammatory one seen in SpA. The long half-life of Secukinumab (24-31 days) presents challenges when considering treatment with other biologics, such as anti-TNFs, which may cause excessive immunosuppression.

**Image:**

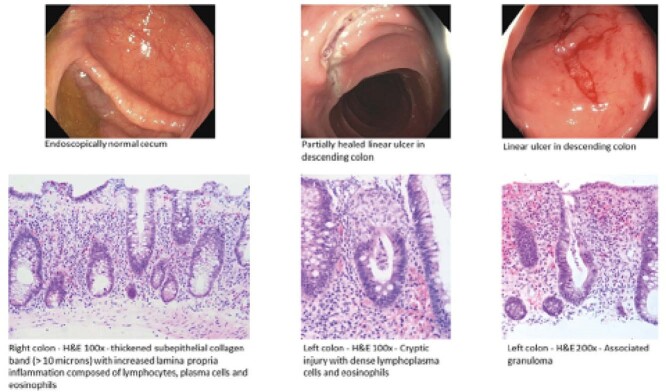

**Conclusion(s):**

New onset colitis is a described but rare entity in patients treated with Secukinumab presenting several diagnostic and treatment challenges.

**Please acknowledge all funding agencies by checking the applicable boxes below:**

None

**Disclosure of Interest:**

None Declared

